# Does Encapsulation Improve the Bioavailability of Polyphenols in Humans? A Concise Review Based on In Vivo Human Studies

**DOI:** 10.3390/nu16213625

**Published:** 2024-10-25

**Authors:** Ali Ali Redha, Chamali Kodikara, Daniel Cozzolino

**Affiliations:** 1 The Department of Public Health and Sport Sciences, University of Exeter Medical School, Faculty of Health and Life Sciences, University of Exeter, Exeter EX1 2LU, UK; 2 Centre for Nutrition and Food Sciences, Queensland Alliance for Agriculture and Food Innovation (QAAFI), The University of Queensland, Brisbane, QLD 4072, Australia; d.cozzolino@uq.edu.au; 3 Department of Food & Human Nutritional Sciences, University of Manitoba, Winnipeg, MB R3T 2N2, Canada; kodikarc@myumanitoba.ca; 4 Canadian Grain Commission, 303 Main St Suite 1000, Winnipeg, MB R3C 3G8, Canada

**Keywords:** bioavailability, encapsulation, delivery systems, intestinal absorption, microencapsulation, nanoencapsulation, phytochemicals, polyphenols

## Abstract

Background/Objectives: Polyphenols offer an array of health benefits that can contribute to well-being. Nevertheless, their bioactivity can be compromised due to their low bioavailability. Encapsulation has been explored as a strategy to enhance the stability and bioavailability of polyphenols. During encapsulation, polyphenols are protected from degradation by a wall material that acts as a protective coating. This coating shields the polyphenols from the harsh physiological conditions of digestion, ensuring their delivery to the intestine. However, the majority of evidence, particularly regarding bioavailability after digestion, is derived from in vitro studies. While these studies provide valuable preliminary insights, they cannot definitively confirm the effects in vivo due to their inability to accurately replicate physiological conditions and the complex gut microbial ecosystem. Consequently, this review seeks to evaluate the current evidence from in vivo human studies to elucidate the efficacy of encapsulation in improving polyphenols’ bioavailability. Results and conclusions: Current clinical evidence on the impact of encapsulation on polyphenol bioavailability is primarily focused on polyphenols derived from grape pomace, cocoa, and bilberries, as well as individual polyphenols such as fisetin, hesperidin, and curcumin. Encapsulation has been an effective technique in improving the bioavailability of individual polyphenols like hesperidin, fisetin, and curcumin. However, this approach has not yielded consistent results when applied to groups of polyphenols, such as bilberry anthocyanins or cocoa phenolic acids. Encapsulation by micellization has shown promising results in improving the bioavailability of curcumin in a nutraceutical context. Further studies are needed to explore the bioavailability of encapsulated polyphenols, especially in the functional food context.

## 1. Introduction

Fruits and vegetables are recognized as nutritional powerhouses, teeming with a diverse array of bioactive compounds, including invaluable phytochemicals. These bioactive compounds encompass a broad spectrum of chemical classes, with polyphenols, terpenoids, phytosterols, alkaloids, and organosulfur compounds representing some of the most prominent examples. Extensive research has unveiled the remarkable potential of phytochemicals, especially polyphenols, to exert a wide range of beneficial effects on human health [1]. For instance, anthocyanin polyphenols, abundantly found in berries and other fruits, have garnered significant attention for their potent antioxidant, anti-inflammatory, anti-diabetic, and anti-cancer properties [2]. Anthocyanins have been investigated as functional food ingredients to support individuals across a wide spectrum, from those seeking weight management [3] to athletes aiming to enhance athletic performance [4,5]. An estimated daily intake of anthocyanin pigments has been reported to be 12.5 mg per person in the United States [6]. However, the realization of the health benefits of anthocyanins is hindered by a major challenge—their low bioavailability. About 1–2% of anthocyanins can reach the cells to possess their bioactivity [7]. Anthocyanins have low stability at the pH of cells and most biological fluids [6]. They undergo significant chemical transformations during gastrointestinal digestion [8]. In the acidic environment of the stomach, they predominantly exist as flavylium cations. However, within the intestine (alkaline environment), the predominant form is the carbinol, which exhibits reduced absorption. Additionally, anthocyanins undergo phase II metabolic processes (glucuronidation, sulfation, and methylation) as well as enzymatic and microbial catabolism [9]. These transformations result in the formation of various metabolites, including anthocyanin glucuronides, phenolic acids (such as ferulic, caffeic, vanillic, gallic, protocatechuic, syringic, and 4-hydroxybenzoic acids), and aldehydes (phloroglucinaldehyde) [9]. In fact, due to the large size and hydrophilic nature of anthocyanins, these compounds exhibit poor passive diffusion across cellular barriers [10]. Compounding this issue, the labile nature of anthocyanins renders them susceptible to degradation during food processing and storage due to factors such as oxygen, temperature, and pH, further compromising their bioavailability [11].

One of the most promising strategies to enhance the stability and bioavailability of polyphenols involves encapsulation, encompassing both microencapsulation and nanoencapsulation techniques [12]. Encapsulation aims to protect within a matrix that facilitates controlled release during gastrointestinal digestion. This process involves mechanically and physicochemically entrapping the substance of interest within a coating material to produce particles of varying sizes, ranging from nanometers to millimeters [13]. The encapsulated compound, referred to as the core material, is dispersed within a matrix known as the coating or shell. This approach utilizes a diverse range of wall materials with varying properties to create protective barriers around the phytochemicals. A wealth of research is dedicated to exploring the efficacy of different wall materials, including various gums, proteins, and even underutilized agricultural by-products, in optimizing encapsulation efficiency [14,15,16]. The resulting encapsulated powders can be seamlessly integrated into food products, where the protective encapsulation safeguards phytochemicals from degradation during processing and storage [17]. Moreover, these encapsulated extracts hold immense potential as nutraceuticals, offering consumers a convenient means to supplement their diets with concentrated phytochemicals [18]. However, the extent to which encapsulation can truly enhance the bioavailability of phytochemicals during the complex process of digestion remains a subject of ongoing investigation and debate.

The impact of encapsulation on polyphenols’ bioaccessibility and bioavailability has been extensively investigated using in vitro gastrointestinal models [19,20,21,22,23]. While these models offer good preliminary insights, their limitations restrict their ability to accurately predict in vivo outcomes [24]. The human gastrointestinal tract is a dynamic environment characterized by continuous food breakdown; enzyme release; and complex biochemical interactions influenced by physiological factors such as temperature, pH, and hormonal regulation, which are not considered in vitro models. Moreover, the majority of in vitro studies overlook or simplify the lower gastrointestinal tract, including the colon, which harbours a diverse microbiota crucial for polyphenol metabolism. The absence of this microbial ecosystem can significantly distort bioavailability estimates [25], as a vast array of polyphenols undergo microbial transformations such as hydrolysis, reduction, oxidation, and conjugation [26] before absorption. Compounding this complexity, the gut microbiota composition exhibits substantial interindividual variability influenced by factors like sex, physiology, lifestyle, and diet [27]. Finally, most studies exclusively assess bioaccessibility, neglecting the subsequent intestinal absorption phase essential for determining bioavailability. Consequently, while in vitro models provide valuable predictive information about the potential effects of encapsulation on polyphenol fate, evidence from human clinical trials remains indispensable for comprehensively understanding the impact of encapsulation on bioavailability.

Therefore, the primary objective of this review is to evaluate the impact of encapsulation on the bioavailability of polyphenols in humans as determined through clinical trials. By examining the available evidence, this review aims to elucidate the extent to which encapsulation technologies can enhance the absorption and utilization of these bioactive compounds in the human body. Ultimately, this research seeks to provide a comprehensive understanding of the efficacy of encapsulation strategies in optimizing the delivery and bioavailability of polyphenols for potential health benefits.

## 2. Bioavailability of Polyphenols

Bioavailability represents the fraction of a nutraceutical or bioactive compound that successfully traverses the gastrointestinal tract, undergoes metabolic processes, and is subsequently distributed to target organs and tissues [28]. It is affected by bioaccessibility, absorption, and transformation of the compound of interest [29]. The bioavailability of polyphenols is typically assessed by quantifying the concentration of the polyphenol/phenolic compound of interest and its metabolites in blood plasma and/or the metabolites in urine. In general, the methodologies employed to evaluate the bioavailability of polyphenols have exhibited some variability across studies, potentially influencing the comparability of findings. For example, some studies may prioritize the area under the curve (AUC) of a polyphenol’s plasma concentration-time profile, which represents the total exposure to the compound. Others may consider the maximum plasma concentration (C_max_), indicating the peak level achieved. Additionally, some investigations focus on the excreted concentration of polyphenol metabolites, providing insights into metabolic pathways and elimination rates.

In this concise narrative review, we have identified and evaluated the clinical trials (human studies) of various study designs that have considered supplementing their participants with any form of encapsulated phenolic, polyphenolic, or (poly)phenolic compounds. The literature search was conducted in Scopus, PubMed, Web of Science, Google Scholar, and Embase to identify relevant studies published in English. The search was initially conducted in July 2023 and updated in October 2024. A limit in terms of year of publication was not applied. The clinical research investigating the bioavailability of encapsulated phytochemicals is still in its infancy. To date, the available studies have been remarkably limited in scope, focusing primarily on polyphenols. A summary of the current studies is shown in Table 1.

### 2.1. Grape Pomace Polyphenols

Grape pomace, a byproduct of winemaking, comprises grape skins, seeds, and stems. This material is a rich source of polyphenols, including phenolic acids (hydroxybenzoic and hydroxycinnamic acids), flavan-3-ols, flavonols, anthocyanins, proanthocyanidins, and resveratrol [41]. This by-product can be utilized as a functional food ingredient in order to enhance the bioactive potential of food products, including their antioxidant activity, while reducing food waste.

Grape pomace polyphenols have been nano-encapsulated using zein and a basic amino acid (a zein/phenolic extract mass of 3/1 (*w*/*w*)) by spray-drying and incorporated into red wine (dealcoholized) [30]. The effect of nano-encapsulated polyphenols was compared to both free phenol-enriched and control wines without added polyphenols in a human study. The C_max_ and AUC values of plasma phenolic acids, stilbenes, flavan-3-ols, phenyl alcohols, and anthocyanins revealed no significant difference between nano-encapsulated and non-encapsulated conditions, indicating that nano-encapsulation did not affect the rate or extent of absorption. In fact, the AUC of resveratrol sulphate (a stilbene) and malvidin-3-*O*-glucoside (an anthocyanin) was significantly lower (*p* < 0.05) in the nano-encapsulated condition compared to the non-encapsulated sample. Given the extensive metabolism of polyphenols, urine phenol excretion is generally a more reliable indicator of bioavailability. Among more than 20 polyphenolic metabolites analysed, only syringic acid salts and malvidin-3-*O*-glucoside were excreted significantly greater (*p* < 0.05) in the encapsulated condition. Overall, this encapsulation method did not effectively enhance the bioavailability of most grape pomace polyphenols. It is also crucial to note that sample size was determined by reference to previous studies, rather than through a priori power analysis.

### 2.2. Cocoa polyphenols

Cocoa is derived from the cacao (*Theobroma cacao* L.) bean, which contains 6–8% of (poly)phenol content (by dry weight) [42]. Catechins are the primary polyphenols (33–42%) found in cacao beans, followed by leucocyanidins (23–25%) and anthocyanins (5%) [43]. Like other polyphenols, those found in cocoa can degrade during food processing. Consequently, the (poly)phenol content of chocolate may be reduced. Added to that, the low stability of polyphenols during digestion also contributes to low bioavailability. Thus, encapsulating cocoa polyphenols in food products (especially chocolate formulations) could be a good approach to conserving the phytochemicals.

Cocoa-nut cream containing partially microencapsulated cocoa polyphenol extract, nano-complexed with gastric-resistant maize starch, has been developed, and its effect on the bioavailability of phenolic acids and flavonoids in humans has been investigated [31]. The bioavailability of phenolic acids and flavonoids was compared with cocoa-nut cream containing cocoa (poly)phenol extract in free form and cocoa-nut cream without extract. With respect to phenolic acids, they initially showed a higher serum concentration compared to encapsulated phenolic acids with a maximum concentration of ≈198 nmol/L after 30 min of ingestion. Encapsulation resulted in a significant decrease in total phenolic acid concentration in serum compared to free phenolic acids and the control (726.8 nmol vs. 1954.3 nmol and 1459.4 nmol, respectively) (*p* < 0.05). In addition, encapsulation resulted in the lowest excretion of phenolic acids in both the first 6 h and overall 24 h compared to the other conditions. With respect to flavonoids, encapsulation resulted in a 13.9-fold decrease in AUC_0–6_ for (epi)catechin compared to free flavanols. Moreover, encapsulation also significantly decreased the excretion of flavanols in urine (*p* < 0.05). Overall, encapsulation significantly reduced the bioavailability of cocoa phenolic acids and flavanols in this study. On the other hand, free flavanols and phenolic acids were more readily absorbed and excreted. A critical limitation of this study is the lack of characterization of the encapsulated cocoa polyphenol extract, preventing a clear understanding of whether it was micro- or nano-encapsulated. Consequently, determining the reasons for the encapsulation’s failure to enhance bioavailability is unobtainable.

### 2.3. Orange Flavanone

**Figure 1:**
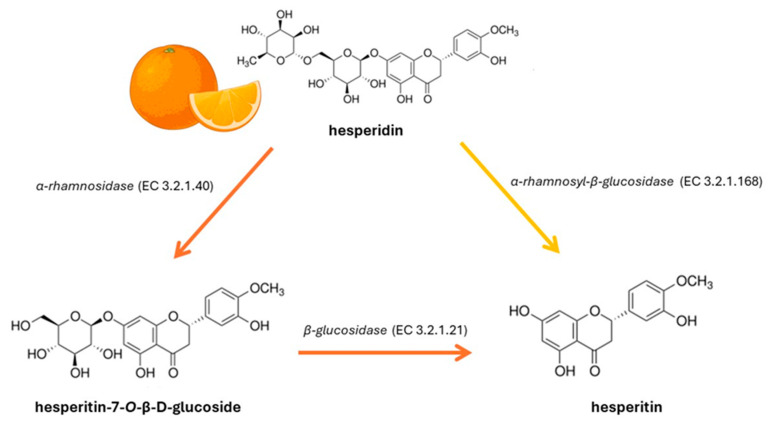
Enzymatic pathways for hesperidin conversion to hesperetin.

Oranges, along with other citrus fruits, are rich sources of hesperidin, a valuable flavanone compound. To be biologically active, hesperidin undergoes a transformation process known as deglycosylation. This process involves the removal of sugar molecules from the hesperidin structure [44]. The primary enzyme responsible for this conversion is α-rhamnosyl-β-glucosidase (αRβGl), which directly converts hesperidin into hesperetin (Figure 1). Alternatively, hesperidin can be sequentially transformed into hesperetin-7-O-glucoside by α-rhamnosidase, followed by further deglycosylation to hesperetin through the action of β-glucosidase (Figure 1) [44].

A study by Tomás-Navarro et al. (2014) explored the effectiveness of encapsulating hesperidin with gum arabic in improving the bioavailability [32]. Orange hesperidin was encapsulated by coacervation with gum arabic as an approach to improve the solubility of the formulation in aqueous media. The bioavailability of hesperitin (after consumption of 89.1 mg in 200 mL of juice), in terms of total excreted equivalents in 24 h, increased significantly after consumption of an encapsulated hesperidin beverage (4.8%) in comparison to free hesperidin (2.2%). Gum arabic, also known as gum acacia, is made up of glycoproteins and polysaccharides (mainly polymers of arabinose and galactose) [45]. The interaction between hesperidin functional groups and the components of gum arabic needs investigation to understand the encapsulation mechanism involved. It is also crucial to note that the amount of hesperitin excreted by individuals can vary significantly [32]. Therefore, future research might benefit from measuring hesperitin levels in plasma to assess bioavailability.

### 2.4. Fisetin

**Figure 2:**
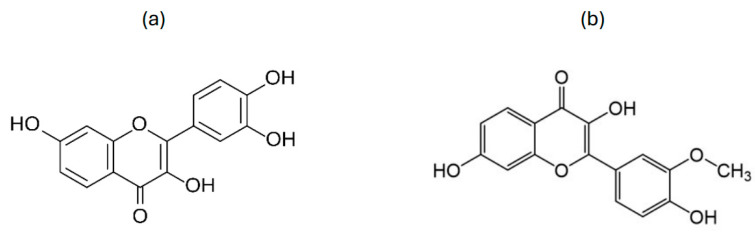
Chemical structure of (**a**) fisetin and (**b**) geraldol.

Fisetin is a flavonol (Figure 2a) present in various fruits and vegetables such as strawberries, apples, persimmons, and cucumbers, with concentrations ranging between 2 and 160 μg/g [46]. Fisetin is capable of suppressing tumour growth, inducing cell death, and counteracting oxidative stress [46]. However, fisetin has a low water solubility (10.45 g/mL) and high lipophilicity (logP 3.2), which contribute significantly to its low bioavailability (44.1%) [47]. Fisetin undergoes methylation by the enzyme methyltransferases, in the liver, to yield geraldol (Figure 2b) [48]. It has been proposed that catechol-O-methyl transferase (COMT) is responsible for the methylation step due to the presence of a catechol ring in fisetin.

Fisetin has recently been encapsulated into fenugreek galactomannan hydrogel scaffolds [33]. Encapsulated fisetin had a reduced crystallinity compared to free fisetin. It had a spherical morphology with a smooth translucent surface. In terms of particle size, the encapsulated product had a larger particle size (151.6 ± 5.1 nm) due to increased hydrodynamic volume from galactomannan chains. The pharmacokinetics of encapsulated fisetin was compared with free (unformulated) fisetin in healthy individuals [33]. Consumption of encapsulated fisetin resulted in significantly higher plasma fisetin levels compared to free fisetin (AUC_0–12 h_ = 341.4 vs. 12.67 ng·h/mL). In addition, encapsulation increased the C_max_ of fisetin by more than 23 times in comparison to free fisetin (238.2 vs. 9.97 ng/mL). Nevertheless, the study did not report the percentage bioavailability of fisetin. Instead, it evaluated the pharmacokinetics of geraldol, the metabolite of fisetin. Similar to fisetin, the level of plasma geraldol from microencapsulated fisetin was 11.1-fold greater than gerladol from free fisetin (227.14 vs. 20.48 ng·h/mL), and the C_max_ was 10 times higher. The time to reach peak geraldol concentration (t_max_) was slightly delayed with encapsulated fisetin, and the half-life of geraldol was longer. Overall, the combination of micelles and hydrogel created a “natural self-emulsifying reversible hybrid-hydrogel system” (N-SERH) that enhanced fisetin absorption. The hydrogel’s hydrophilic nature and hydrogen bonding can contribute to the stability and size of the released micelles.

### 2.5. Bilberries Anthocyanins

**Figure 3:**
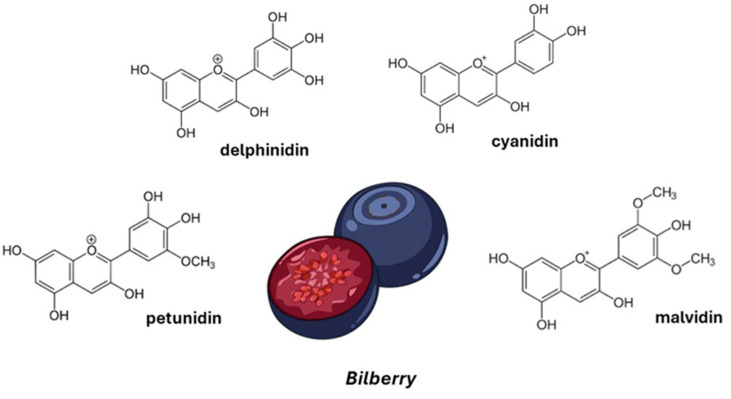
Bilberry fruit and the chemical structure of the backbone of the main anthocyanins in bilberry.

Bilberries (*Vaccinium myrtillus* L.) are berries native to northern Europe. They are rich in anthocyanins, such as delphinidins (15.17%), cyanidins (8.36%), petunidins (6.64%), and malvidins (5.43%) (Figure 3), which are particularly concentrated in the bilberry skin. Owing to their remarkable antioxidant properties, anthocyanins have been explored for a variety of applications, including cardiovascular health, brain function, weight management [49], and athletic recovery [7]. However, anthocyanins are susceptible to degradation from factors such as pH, temperature, and oxygen exposure. These conditions, prevalent in food processing and the human digestive system, significantly reduce their bioavailability. For instance, the intact flavylium cation present in bilberry is expected to undergo ring cleavage and degradation during the gastrointestinal passage.

A study by Mueller et al. (2018) explored how encapsulation strategies affect anthocyanin bioavailability and intestinal accessibility using bilberry extract [34]. Bilberry extract was microencapsulated with (i) whey protein isolate by emulsification and thermal gelation or (ii) highly esterified citrus pectin by spray-drying. The investigators assessed the breakdown of anthocyanins in urine, plasma, and ileal effluent from both healthy people and ileostomists. Microencapsulation with whey protein reduced the total anthocyanin content in ileal effluents by 10% compared to the free extract. In plasma, a 28% decrease in anthocyanins and a 6% decrease in degradation products were detected (healthy individuals). Conversely, urine samples showed a 108% increase in anthocyanins and a 48% increase in degradation products. This suggests that whey protein was not effective in stabilizing bilberry anthocyanins. However, our results showed that whey protein capsules did not stabilize anthocyanins and may have been absorbed in the stomach, resulting in a high concentration of anthocyanins and their degradation products in the urine. On the other hand, the encapsulating approach employing citrus pectin had a completely different effect—it helped in increasing intestinal accessibility during the passage through the small intestine (by 24%). Although microencapsulation using pectin increased the intestinal concentrations of anthocyanins, it did not enhance the absorption of anthocyanins and thus did not affect bioavailability. Unexpectedly, encapsulation procedures were found to alter PGAL production, a point that may be important in understanding the larger health implications of anthocyanins. Overall, the encapsulation process did not significantly enhance the overall bioavailability of anthocyanins [34].

To date, research on the impact of microencapsulation on anthocyanin bioavailability remains limited, with only one clinical study conducted thus far. While this single study, a pilot in nature, is insufficient to draw definitive conclusions, it does offer preliminary insights. Given the inherent instability of anthocyanins, a compound class known for its susceptibility to degradation, exploring encapsulation technologies as a potential stabilization and bioavailability enhancement strategy remains questionable and warrants further investigation.

### 2.6. Curcumin

**Figure 4:**
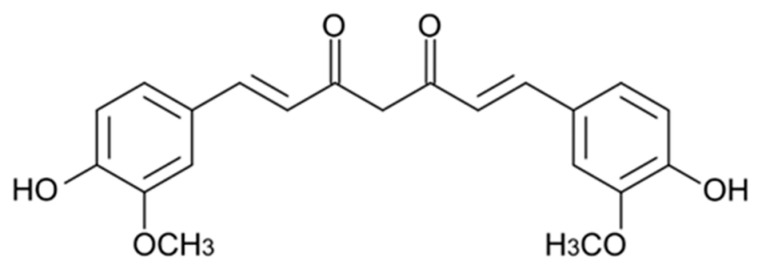
Chemical structure of curcumin.

Curcumin is a diarylheptanoid polyphenol (Figure 4) and the primary active constituent of the turmeric (*Curcuma longa*) rhizome [50]. Curcumin is a lipophilic molecule (water solubility of <8 μg/mL) with low permeability that contributes to limiting its intestinal absorption during digestion [51,52]. In addition, it is extensively metabolized by the intestine and undergoes rapid first-pass metabolism (short elimination half-life of <2 h), reducing the amount of curcumin reaching the cells to exhibit its bioactive potential [53]. The bioavailability of curcumin is less than 1% (0.05 μg/mL) [52].

Curcumin-containing curcuminoid extract has been encapsulated using cellulose derivative (first layer) and hydrogenated vegetable oil (external layer) through a double-coating approach [35]. In a functional food context, encapsulated curcumin was then incorporated into bread (ECB, containing 72.7% of curcuminoids), and its bioavailability was compared with bread containing free curcumin (FCB, containing 95% pure curcuminoid extract from turmeric) and bread containing encapsulated curcumin combined with additional polyphenols (piperine, quercetin, and genistein, 1% of each) (ECBB, containing 66.5% of curcuminoids), which have been reported [54] to improve curcumin bioavailability (in vitro). During a 24 h period, the researchers examined for parental (unchanged) and metabolized curcuminoids and phenolic acids in the blood, urine, and faeces of their human participants. The quantities of curcuminoids in the plasma were consistently less than 4 nmol/L, and their glucuronide forms were much lower, indicating inadequate absorption. Curcumin encapsulation significantly delayed and increased absorption when compared to the free form, showing that the encapsulation technique has the potential to maximize curcumin bioavailability. The blood and urinary concentrations of phenolic acids, such as ferulic and vanillic acid, were found to be significantly greater (between 2- and 1000-fold) than those of curcuminoids, with ECBB-enriched bread having the greatest amounts. Curcuminoids were found to be six times more plentiful in faeces after eating ECB-enriched bread than FCB-enriched bread. Furthermore, phenolic acids in faeces were four times higher after ECBB consumption than after ECB consumption. The findings suggest that encapsulating curcumin not only boosts its bioavailability when added to enriched bread but that it may also prevent its rapid biotransformation. It is also important to highlight that the addition of polyphenols did not improve the bioavailability of curcumin. This could be due to competitive absorption taking place at the intestinal mucosa between curcumin and the other polyphenols, resulting in a delay in curcumin absorption and enhanced degradation at the intestinal lumen [35]. Overall, encapsulated curcuminoids, with or without polyphenols, increased serum curcuminoids by 7.3 and 4.6 times, respectively, compared to free curcuminoids. However, co-encapsulating curcuminoids with polyphenols did not improve bioavailability compared to encapsulated curcuminoids alone. In fact, serum curcuminoids were 63% higher after consuming encapsulated curcuminoids without polyphenols.

In a nutraceutical context, curcumin has been nano-encapsulated using alginate polysorbate 80 nanoparticles to increase its water solubility and dispersibility [36]. Healthy males were given 100 mg doses of either nano-encapsulated or free curcumin as suspensions in a drink to compare the oral bioavailability of the two formulations. The nano-encapsulated curcumin exhibited a more rapid onset of action, reaching a maximum plasma concentration (C_max_) at 2 h compared to 4 h for the free curcumin. The oral bioavailability of the nano-encapsulated curcumin was significantly greater than free curcumin (*p* < 0.01) by 5-fold. Thus, polysorbate 80 can be considered a potential encapsulating agent for curcumin as it is a potent emulsifier and can solubilize curcumin by forming mixed micelles shielding the lipophilic curcumin (hydrophobic core) while the outer hydrophilic shell protects and stabilizes the molecule in aqueous environments through digestion [55]. Nevertheless, this study [36] needs to be interpreted with care since it is associated with a high risk of bias. The study design was not clearly described, including details on randomization, participant blinding, and investigator blinding.

Another study [40] focusing on curcumin in nutraceutical formulations investigated the bioavailability of curcumin in different commercial supplements that were based on encapsulation or complexation of curcumin with various agents. These formulations aimed to increase the bioavailability of curcumin either through enhancing post-digestive stability and solubility of curcumin or improving its post-absorptive processes such as inhibition of biotransformation. The bioavailability of native curcuma extract, liposomal curcumin, curcuma extract with turmeric oils, curcuma extract with adjuvants, submicron-particle curcumin, phytosomal curcumin, curcumin-*γ*-cyclodextrin complex, and micellar curcumin in healthy adults was assessed and compared. Curcumin plasma AUC_0–24 h_ of micellar and *γ*-cyclodextrin complex formulation was significantly (*p* < 0.05) higher than other formulations. In comparison to free curcumin (native curcuma extract), micellar and *γ*-cyclodextrin curcumin had a 57-fold and 30-fold improved bioavailability. In fact, micellar curcumin had a significantly greater bioavailability in comparison to curcumin-*γ*-cyclodextrin. Based on the findings of this study, encapsulation strategies targeting increasing curcumin water-solubility seem to be more effective in comparison to those strategies considered post-absorptive action (e.g., inhibition of biotransformation or efflux transporters). Similarly, three other studies investigating the in vivo bioavailability of micellar curcumin also reported a significant increase in curcumin bioavailability, at 185-fold (410 mg curcumin supplementation) [37], 88-fold (80 mg curcumin supplementation) [38], and 14-fold (35 mg curcumin supplementation) [39], in comparison to free curcumin, based on plasma AUC. Thus, based on the current evidence from several in vivo human studies [36,37,38,39,40], micellization can be considered an effective encapsulation technique to improve the bioavailability of curcumin. Most of these studies have used polysorbates (such as Tween-80), which are nonionic surfactants, for curcumin micellization. Polysorbates/Tween comprises a non-polar fatty acid group that is esterified to a polar polyoxyethylene sorbitan group. Curcumin has a solubility of 34.38 ± 0.78 mg/mL in Tween-80 [56]. Curcumin binds to polysorbates through hydrophobic and electrostatic interactions [57]. A study comparing binding curcumin with Tween 20, 60, and 80 reported that Tween 60 micelles create the most hydrophobic environment for curcumin due to their longer, saturated alkyl chains [58]. This leads to a higher p*K*_a1_ and stronger binding affinity compared to Tween 20 and Tween 80. The findings of this study [58] suggest that increasing the length of the alkyl chain in Tween surfactants enhances hydrophobic binding with curcumin, while unsaturation has the opposite effect. Future studies may focus on Tween 60 for curcumin micellization.

## 3. Limitations and Future Perspectives

Polyphenols exhibit diverse solubility and stability profiles within the gastrointestinal tract, influenced by factors such as pH, enzymes, and bile acids, ultimately impacting their bioaccessibility and bioavailability. Encapsulation has demonstrated potential in enhancing the bioavailability of specific bioactive polyphenols like hesperidin, hesperetin, fisetin, and curcumin. However, inconsistent results have been reported for complex polyphenol mixtures from sources such as grape pomace, cocoa, and bilberries. While definitive conclusions are challenging due to limited studies, a discernible trend suggests that encapsulation is more effective in improving the bioavailability of individual-targeted concentrated polyphenols compared to complex polyphenol mixtures. This is plausible because the determined encapsulation efficiency of mixed polyphenols is likely influenced by differential interactions with encapsulation materials, potentially leading to overestimated overall encapsulation values. In contrast, focusing on specific polyphenols provides a more accurate assessment of encapsulation efficacy. Thus, future research aimed at stabilizing polyphenols could prioritize optimizing encapsulation processes for a key representative polyphenol from the chosen source. In addition, future studies should also clearly report the wall material-to-core material ratio and perform optimizing experiments to ensure maximal encapsulation efficiency. In silico molecular docking studies can provide valuable insights into the interactions between wall materials and compounds of interest, revealing the molecular mechanisms underlying different types of binding. Thus, such strategies are useful to be considered alongside encapsulation studies.

In addition, the effectiveness of encapsulation on polyphenol bioavailability should be evaluated differently based on the intended application. For individual polyphenols with established pharmaceutical potential, such as curcumin, resveratrol, and genistein, direct administration of encapsulated products to participants in clinical trials is appropriate. However, when investigating polyphenol blends from diverse food sources with less characterized bioactivity, a food-based approach is necessary. Encapsulated polyphenols should be incorporated into food products to assess their bioavailability within a real-world dietary context. This strategy is crucial for developing functional foods enriched with these polyphenol blends. In addition, with respect to this context, matrix effects must be considered when evaluating the impact of encapsulation on polyphenol bioavailability. In functional foods, the interaction between the encapsulated polyphenols and the food matrix is more complex compared to nutraceuticals, where the compound is typically delivered as a powder or capsule. The increased processing in functional foods can influence polyphenol bioavailability. In addition, fiber, proteins, and fats of the food matrix can interact with polyphenols, affecting their release and absorption [29]. When evaluating encapsulated polyphenols as functional food ingredients, it is crucial to meticulously determine the optimal extract-to-wall material ratio and compound concentration. A low concentration of encapsulated extract within the functional food could necessitate the addition of excessive quantities, potentially leading to challenges related to the physicochemical properties and sensory attributes of the final product.

Prior to evaluating an encapsulated sample for clinical trials, it is essential to consider certain practices for conducting experiments to a better understanding of bioavailability. Thorough characterization of encapsulated products prior to clinical trials is essential but often overlooked. By examining parameters like particle size, zeta potential, surface morphology, swelling capacity, solubility, acidity, and pH stability, researchers can establish correlations between the physicochemical properties of the encapsulated material and its pharmacokinetic behavior. Understanding these relationships is crucial for predicting the product’s fate during digestion and absorption.

Future clinical trials investigating the bioavailability of encapsulated phytochemicals must prioritize rigorous methodological design to mitigate the risk of bias, a common shortcoming in previous studies. Implementing randomized allocation of participants to intervention groups will ensure equitable distribution of confounding factors. Maintaining participant and assessor blindness to treatment conditions can significantly reduce subjective bias. Additionally, accurate estimation of effect sizes is crucial for determining appropriate sample sizes, enhancing the study’s power to detect meaningful differences in bioavailability between encapsulated and free phytochemicals. In addition, careful dose selection is important in clinical trials involving encapsulated polyphenol blends. The individual concentrations of different polyphenols can be relatively low compared to a high dose of a specific encapsulated polyphenol, potentially limiting the observed effects on bioavailability. Consequently, insufficient dosing may hinder the detection of statistically significant positive or negative changes.

It is important to highlight that encapsulated polyphenols are the main class of phytochemicals whose bioavailability has been studied in humans. While other phytochemicals like terpenes (e.g., carotenoids) and organosulfur compounds (e.g., isothiocyanates) have also been encapsulated, their bioavailability in humans remains largely unexplored. Thus, the scope of this review is constrained by the current availability of clinical studies in this research field.

## Figures and Tables

**Table 1:** Summary of clinical-based evidence on the influence of encapsulation/complexation on the bioavailability of different polyphenols.

Polyphenol	Encapsulation Method	Study Type	Participants	Intervention	Key Outcomes	Ref.
Grape pomace polyphenols	Nanoencapsulation of extract in nanoparticles with a zein (maize protein) matrix and a basic amino acid by spray-drying. Encapsulated product was incorporated in dealcoholized red wine.	Randomized, controlled, crossover trial with three treatment conditions	N = 12; healthy volunteers (6 males and 6 females), age range: 19–50 years, mean BMI of males: 26.4 kg/m^2^, mean BMI of females: 26.1 kg/m^2^.	Participants consumed (a) 100 mL of dealcoholized red wine; (b) dealcoholized red wine with non-encapsulated phenolic extract (1.3 g phenolic extract); and (c) 100 mL of dealcoholized red wine with nano-encapsulated phenolic extract (9 g and containing 1.3 g of phenolic extract).	Nano-encapsulation of extract slightly, but significantly, increased the bioavailability of malvidin-3-*O*-glucoside (most abundant grape anthocyanin) determined in urine. Urine molar exertion of encapsulated malvidin-3-*O*-glucose was 0.14 µmols/24 h, while that of non-encapsulated and control were 0.08 and 0.06 µmols/24 h, respectively. Yet, did not show a positive effect on the bioavailability of most polyphenols.	[30]
Coca polyphenols	Partial microencapsulation based on the formation of nano-complexes with a high-amylose maize starch as a coating agent by spray-drying. The encapsulated product was incorporated in cocoa-nut cream.	Single-blind, randomized, cross-over trial with three treatment conditions	N = 12; healthy volunteers (4 males and 8 females), mean age: 24 years, mean BMI: 23.1 kg/m^2^.	Participants consumed three portions (99 g) of cocoa-nut cream (daily intake of 12 µmol of phenolic acids and 190 µmol of flavanols) and cocoa-nut cream enriched with a 1.5% (*w*/*w*) cocoa polyphenol extract (daily intake of 28 µmol of phenolic acids and 385 µmol of flavanols) in free and encapsulated forms.	Encapsulation resulted in a significant 13.9-fold decrease in AUC_0_–_6_ for serum (epi)catechin compared to free flavanols. A significant decrease in total phenolic acids in encapsulated form was determined during 0–6 h compared to free from (890 vs. 11,400 nmol).	[31]
Orange flavanone (hesperidin)	Hesperidin formulation was encapsulated by coacervation using gum arabic. Encapsulated product was added to a coloured and non-flavanone-containing beverage.	Crossover study (no further details specified)	N = 18; healthy volunteers (10 males and 8 females), age range: 25–50 years.	Participants consumed 200 mL of test beverage containing 90 mg hesperetin equivalent of (a) free and (b) encapsulated formulation ^†^.	Encapsulation increased the bioavailability of hesperidin between 2 and 3 times in comparison to the free hesperidin formulation.	[32]
Fisetin	Fisetin micelles were encapsulated into a fenugreek galactomannan hydrogel scaffold.	Double-blind, randomized, controlled, crossover trial	N = 15; healthy volunteers (12 males and 3 females), age range: 22–55 years.	Participants consumed 1000 mg of unformulated fisetin or 1000 mg of encapsulated fisetin (delivering 192 mg of fisetin).	Encapsulation increased the plasma concentration of fisetin by 26.9-fold (based on AUC values). Encapsulation also increased the maximum plasma concentration (C_max_) by 23 times.	[33]
Bilberry (*Vaccinium myrtillus* L.) anthocyanins	Bilberry extract was microencapsulated with whey protein isolate by emulsification and thermal gelation.	Pilot study (no further details specified)	N = 10; five healthy female volunteers (mean age: 33 years, mean BMI: 23 kg/m^2^) and five female ileostomist volunteers (mean age: 41 years, mean BMI: 27 kg/m^2^).	Participants consumed equimolar amounts of anthocyanins, either as a single bolus of (a) non-encapsulated extract (10 g), (b) extract encapsulated with whey protein (144 g), or (c) extract encapsulated with citrus pectin (30 g).	Microencapsulation with whey protein decreased the total anthocyanin content in the ileal effluents by 10% compared to free extract and resulted in 28% less anthocyanins and 6% less degradation products in the plasma of healthy volunteers. However, 108% more anthocyanins and 48% more degradation products were detected in urine.	[34]
	Bilberry extract was microencapsulated with highly esterified citrus pectin by spray-drying.				Microencapsulation could increase intestinal levels of anthocyanins (≈24%) but did not trigger the absorption and thus did not enhance bioavailability.	
Curcumin	Curcuminoid extract (containing 79% curcumin, 19% DMC, and 2% BDMC) was encapsulated by double coating (fluidized bed spray coating, followed by bottom spray) using cellulose derivative as a first layer and hydrogenated vegetable oil as an external layer. Another encapsulated sample was prepared with the addition of piperine, quercetin, and genistein to the extract (i.e., co-encapsulation). Encapsulated products were incorporated into classical bread.	Double-blind, randomized, crossover trial with three treatment conditions	N = 10; healthy volunteers, mean age: 31 years, mean BMI: 23.5 ± 1.2 kg/m^2^.	Participants consumed two classical bread portions containing 1 g of (a) free curcuminoids; (b) encapsulated curcuminoids; and (c) encapsulated curcuminoids with piperine, quercetin, and genistein in a 100 g portion.	Encapsulation significantly increased the bioavailability of curcumin. An increase of 7.3- and 4.6-fold in serum curcuminoids was determined after consumption of bread containing encapsulated curcuminoids and bread containing encapsulated curcuminoids + polyphenols, respectively, compared to free curcuminoids. Co-encapsulation of curcuminoids and polyphenols did not improve bioavailability in comparison to encapsulation without polyphenols. Serum curcuminoids were 63% higher after consumption of encapsulated curcuminoids without polyphenols.	[35]
	Curcumin was encapsulated in alginate-polysorbate 80 nanoparticles by the ionotropic gelation technique.	No clear design specific (two parallel treatment conditions)	N = 6; healthy male volunteers, mean age: 25 ± 3 years, mean BMI: 23.8 ± 1.8 kg/m^2^.	Participants (N = 3) consumed (a) 150 mL of curcumin suspension containing 100 mg of curcumin or (b) 150 mL of curcumin nanosuspension containing 100 mg of curcumin.	Nanoencapsulation increased the oral bioavailability of curcumin by 5-fold in comparison to free curcumin.	[36]
	Curcumin liquid micelles ^a^ mixed into 50 g woodruff syrup.	Randomized, controlled, crossover trial with three treatment conditions	N = 23; healthy volunteers (10 males and 13 females), age range: 20–28 years, BMI 18.5–24.9 kg/m^2^.	Participants consumed 500 mg of curcuminoids (containing 410 mg curcumin, 80 mg DMC, and 10 mg BDMC) as a native powder containing free curcumin or curcumin liquid micelles ^†^.	Curcumin micelles significantly increased the oral bioavailability of curcumin by 185-fold in comparison to free curcumin (based on plasma AUC_0–24 h_).	[37]
	Curcumin micelles ^a^ containing 7% of curcuminoid powder (equal to 6% curcumin) and 93% Tween-80. Curcumin micelles were also prepared with the addition of polyphenols (sesamin, ferulic acid, naringenin, and xanthohumol).	Single-blind, randomized, controlled, crossover trial with four treatment conditions	N = 24; healthy volunteers (N =12, 6 males and 6 females; age 18–35 years, N = 11, 6 males and 5 females; >60 years).	Participants consumed 98 mg of curcuminoids as a native powder containing free curcumin or curcumin micelles capsules (with or without polyphenols) ^†^.	Curcumin micelles significantly increased the oral bioavailability of curcumin by 88-fold, and by 73-fold with the presence of polyphenols, in comparison to free curcumin (based on plasma AUC_0–24 h_). There was no significant difference between the bioavailability of curcumin micelles with and without polyphenols.	[38]
	Curcumin micelles containing Tween-80 (no further information was provided).	Double-blind, randomized, controlled, crossover trial with two treatment conditions	N = 15; healthy volunteers (9 males and 6 females), mean age: 40.3 ± 9.5 years, mean BMI: 25.0 ± 4.1 kg/m^2^.	Participants consumed 43 mg of curcuminoids (35 mg of curcumin) as a native powder containing free curcumin or curcumin micelle capsules.	Curcumin micelles significantly increased the oral bioavailability of curcumin by 14-fold in comparison to free curcumin (based on plasma AUC).	[39]
	Commercially ^††^ available encapsulated/complexed curcumin was used: liposomal curcumin, phytosomal curcumin, curcumin-*γ*-cyclodextrin complex, and micellar curcumin.	Double-blind, randomized, controlled, crossover trial with eight treatment conditions	N = 12; healthy volunteers (6 males and 6 females), age range: 19–31 years, BMI range: 18.5–24.9 kg/m^2^.	Participants consumed 207 mg of curcumin as native curcuma extract or one of the commercial formulations ^†^. (Note: The formulations were normalized to 207 mg curcumin as 6 capsules, each containing 34.5 mg curcumin.)	A significant increase in plasma AUC of curcumin was only determined for micellar curcumin (57-fold) and the curcumin-γ-cyclodextrin complex (30-fold) in comparison to free curcuma extract.	[40]

Abbreviations—AUC: area under the concentration–time curve. BMI: Body Mass Index. DMC: demethoxycurcumin. BDMC: bis-demethoxycurcumin. ^a^ Produced by AQUANOVA AG, Darmstadt, Germany. Curcumin micelles contained 7% curcumin powder (equivalent to 6% curcumin) and 93% Tween-80 (Kolb, Hedingen, Switzerland). ^†^ One or more formulations were also investigated in the clinical trial but were not relevant to the scope of the current review; thus, they have not been mentioned here. ^††^ Brands used: Liposomal curcumin (Longvida; Verdure Sciences, Noblesville, IN, USA), phytosomal curcumin (Meriva; Indena S.p.A., Milano, Italy), curcumin-*γ*-cyclodextrin complex (Cavacurmin; Wacker Chemie, Munich, Germany), and micellar curcumin (NovaSOL Curcumin; AQUANOVA AG, Darmstadt, Germany).
